# THE FEASIBILITY OF TAICHI WITH MUSIC FOR HEALTH IN INDIVIDUALS LIVING WITH ADRD AND THEIR CAREGIVERS

**DOI:** 10.1093/geroni/igad104.3641

**Published:** 2023-12-21

**Authors:** Yan Du, Emma Blackmon, Christina Smith, Cindy Robles, Sandeep Subramanian, Sue Jee, Kylie Meyer, Penny Roberts

**Affiliations:** UT Health San Antonio, San Antonio, Texas, United States; UT Health San Antonio, San Antonio, Texas, United States; WellMed Charitable Foundation, San Antonio, Texas, United States; WellMed Charitable Foundation, San Antonio, Texas, United States; UT Health San Antonio, San Antonio, Texas, United States; UT Health San Antonio, San Antonio, Texas, United States; Case Western Reserve University, Cleveland, Ohio, United States; Loyola University New Orleans, New Orleans, Louisiana, United States

## Abstract

Caregiving demands associated with cognitive and physical function decline in care recipients (CRs) living with Alzheimer’s disease and related dementias (ADRD) contribute to compromised health and quality of life among ADRD caregivers. In turn, the affected health of caregivers affects the care quality of CR and their health. TaiChi or music is evidenced to be beneficial for the generation population and those with various health conditions. This pilot project assessed the feasibility and preliminary efficacy of an 8-week TaiChi with music (TCM) program on individuals with ADRD (e.g., physical and cognitive function) and their family caregivers (e.g., caregiver distress and quality of life). Outcomes were compared between baseline and post-intervention using paired t-tests. Qualitative interview data exploring the perceptions of participating in TCM program were analyzed using content analysis. Five dyads were enrolled in this study (CR age=77.4±6.8; Caregiver age=62.6±6.7). The overall class attendance rate is 65%. Among the small but diverse sample, adult child care dyads had higher class attendance (93%); spousal care and CR with severe cognitive impairment dyads had lower attendance (46%). Improvements were found in assessed health outcomes for both CR and caregivers, and CR’s physical function significantly improved at 8-week (p=0.016). Qualitative data revealed perceived balance improvements; Caregivers perceived calmness and peace, and expressed their interest in participating in TCM in the future. TCM may be more feasible in ADRD individuals with mild to moderate cognitive impairment. Future research will evaluate the efficacy of this intervention in a larger sample with a longer follow-up period.

